# Supplementary material for: Local Myoelectric Sensing During Human Colonic Tissue Perfusion
Source: Diagnostics (Basel). 2024 Dec 20;14(24):2870. doi: 10.3390/diagnostics14242870 (PMC11675604; doi:10.3390/diagnostics14242870)
Supplement: Supplementary file 1 [file diagnostics-14-02870-s001.zip › diagnostics-3347778-supplementary.pdf]

diagnostics

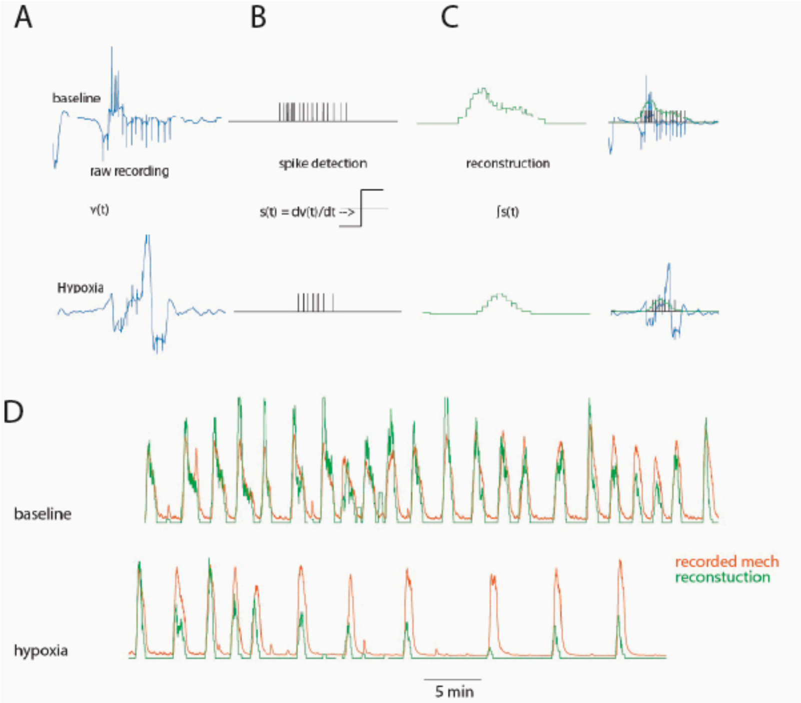

Supplementary Figure S1: Correlation between colonic contraction force and myoelectric activity. (A) Baseline myoelectric activity recorded using the setup described in Figure 2 under normal (top) and hypoxic (bottom) conditions. (B) Filtered spikes. Spikes are filtered by thresholding the derivative of the temporal voltage function. (C) Reconstructed contractions force. The reconstruction was done by convolution of the reconstructed spikes with a rectangular window which estimates the transfer function of the colonic smooth muscle. (D) illustration of the correlation between the actual recorded contractions and the reconstructed contractions over time under normal (top) and hypoxic (bottom) conditions.
